# Supplementary material for: High Adherence to a Mediterranean Alcohol-Drinking Pattern and Mediterranean Diet Can Mitigate the Harmful Effect of Alcohol on Mortality Risk
Source: Nutrients. 2023 Dec 24;16(1):59. doi: 10.3390/nu16010059 (PMC10780794; doi:10.3390/nu16010059)
Supplement: Supplementary file 1 [file nutrients-16-00059-s001.zip › nutrients-2745702-supplementary.pdf]

Supplementary tables

Supplementary Table 1. Baseline Characteristics of Participants by Food Frequency Questionnaire. MICOL/PANEL Castellana Grotte (BA) and NUTRIHEP Studies, Putignano (BA), Italy, 2005-2022

	FFQ	
	Yes	No
N	3411	366
Age at enrollment (yrs) *	50.98 (16.01)	45.56 (15.55)
DBP (mmHg)*	124.98 (16.98)	122.56 (16.27)
SBP (mmHg)*	77.59 (9.39)	77.76 (9.56)
Weight (kg)*	72.98 (14.52)	72.16 (16.83)
BMI (kg/m^2)*	27.13 (4.80)	26.59 (5.54)
Triglycerides (mmol/L)*	1.38 (0.98)	1.27 (0.95)
Total Cholesterol (mmol/L)*	5.12 (1.02)	4.98 (0.96)
HDL (mmol/L)*	1.34 (0.35)	1.34 (0.34)
LDL (mmol/L)*	3.16 (0.87)	3.05 (0.88)
Glucose (mmol/L)*	5.84 (1.34)	5.87 (1.53)
ALT (µkat/L)*	0.27 (0.21)	0.26 (0.19)
Sex***		
Female	1464 (42.9)	188 (51.47)
Male	1947 (57.1)	178 (48.36)
Smoke***		
Never/Former	2823 (82.8)	210 (73.94)
Current	588 (17.2)	74 (26.06)
Education***		
Primary School	1021 (29.9)	44 (18.49)
Secondary School	1061 (31.1)	82 (34.45)
High School	1005 (29.5)	86 (36.13)
Graduated	324 (9.5)	26 (10.92)
Job***		
Managers & Professionals	216 (6.3)	17 (6.30)
Craft. Agricultural and Sales Workers	814 (23.9)	81 (34.03)
Elementary Occupations	799 (23.4)	56 (23.53)
Housewife	415 (12.2)	23 (9.66)
Pensioneers	1164 (34.2)	36 (15.13)
Jobless	216 (6.3)	25 (10.50)
Marital Status***		
Single	561 (16.4)	44 (12.32)
Married/Coupled	2611 (76.5)	129 (36.13)
Separated/Divorced	73 (2.1)	3(0.84)
Widower	166 (4.9)	5 (1.40)

ALT: Alanine Aminotransferase; BMI: Body Mass Index; DBP: Diastolic Blood Pressure; SBP Systolic Blood Pressure; HDL: High-Density Lipoprotein Cholesterol; LDL: Low-Density Lipoprotein Cholesterol; Cells reporting subject characteristics contain * Mean±(SD). ***Number (Percentage) Percentages calculated per column

Supplementary Table 2. Characteristics of Participants by Mediterranean Alcohol Drinking Pattern Score Categories. MICOL/PANEL Studies. Castellana Grotte, (BA), Italy, 2005-2022

	Mediterranean Alcohol Drinking Pattern			
	High (7-9)	Moderate (4-6)	Low (0-3)	*p-value*
N***	1094 (70.5)	398 (25.6)	61 (3.9)	
Age at enrollment (yrs) *	57.69 (14.99)	51.36 (13.24)	55.70 (12.49)	<0.001
DBP (mmHg)*	125.32 (19.44)	123.35 (19.48)	129.97 (20.36)	0.030
SBP (mmHg)*	74.66 (10.14)	76.21 (9.81)	77.60 (9.99)	0.005
Weight (kg)*	75.58 (14.30)	77.87 (14.78)	80.67 (12.15)	0.001
BMI (kg/m^2)*	28.57 (4.72)	28.71 (4.91)	29.12 (3.91)	0.63
Kcal days*	2262.11 (677.61)	2503.60 (702.20)	2855.34 (754.18)	<0.001
Triglycerides (mmol/L)*	1.47 (0.95)	1.60 (1.23)	1.59 (1.15)	0.11
Total Cholesterol (mmol/L)*	5.20 (0.98)	5.29 (1.05)	5.53 (1.07)	0.020
HDL (mmol/L)*	1.31 (0.34)	1.33 (0.34)	1.49 (0.53)	<0.001
LDL (mmol/L)*	3.22 (0.84)	3.24 (0.87)	3.28 (0.94)	0.80
Glucose (mmol/L)*	6.07 (1.37)	6.17 (1.71)	6.27 (1.13)	0.33
ALT (µkat/L)*	0.29 (0.28)	0.30 (0.18)	0.34 (0.15)	0.20
Wine consumption (g alcohol/d) *	18.88 (10.57)	33.29 (27.95)	55.42 (21.09)	<0.001
Beer consumption (g alcohol/d) *	0.83 (2.41)	3.91 (5.18)	14.64 (4.44)	<0.001
Spirit consumption (g alcohol/d) *	0.65 (1.30)	2.12 (3.91)	5.15 (6.84)	<0.001
Sex***				
Female	323 (75.5)	104 (24.3)	1 (0.2)	<0.001
Male	771 (68.5)	294 (26.1)	60 (5.3)	
Smoke***				
Never/Former	933 (74.6)	279 (22.3)	38 (3.0)	<0.001
Current	161 (53.1)	119 (39.3)	23 (7.6)	
CCI**	40 (2-5)	3 (1-5)	4 (3-6)	<0.001
rMED**	8 (7-10)	7 (6-8)	7 (6-8)	<0.001
Age at Death (yrs) **	74.41 (61.06-83.47)	67.32 (56.57-77.52)	72.74 (61.05-78.69)	<0.001
Observation time** (yrs)	16.94 (15.07-17.42)	16.94 (16.13-17.29)	16.93 (14.79-17.30)	0.27
Status***				
Alive and/or Censored	775 (67.7)	329 (28.7)	41 (3.6)	<0.001
Dead	319 (78.2)	69 (16.9)	20 (4.9)	
Cause of Death***				
CVD	100 (81.3)	19 (15.4)	4 (3.3)	0.020
Cr	52 (72.2)	13 (18.1)	7 (9.7)	
DS	36 (65.5)	13 (23.6)	6 (10.9)	
DOC	131 (82.9)	24 (15.2)	3 (1.9)	
Education***				
Primary School	364 (70.1)	137 (26.4)	18 (3.5)	0.40
Secondary School	358 (69.2)	134 (25.9)	25 (4.8)	
High School	249 (70.3)	95 (26.8)	10 (2.8)	
Graduated	123 (75.5)	32 (19.6)	8 (4.9)	
Job***				
Managers & Professionals	40 (59.7)	23 (34.3)	4 (6.0)	0.002
Craft. Agricultural and Sales Workers	246 (63.4)	122 (31.4)	20 (5.2)	
Elementary Occupations	282 (71.0)	105 (26.4)	10 (2.5)	
Housewife	96 (73.8)	26 (20.0)	8 (6.2)	
Pensioneers	415 (76.0)	113 (20.7)	18 (3.3)	
Jobless	15 (60.0)	9 (36.0)	1 (4.0)	
Marital Status***				
Single	98 (67.6)	45 (31.0)	2 (1.4)	<0.001

Married/Coupled	880 (70.2)	322 (25.7)	51 (4.1)
Separated/Divorced	22 (50.0)	18 (40.9)	4 (9.1)
Widower	94 (84.7)	13 (11.7)	4 (3.6)

DBP: Diastolic Blood Pressure; SBP: Systolic Blood pressure; BMI: Body Mass Index; HDL: High-Density Lipoprotein Cholesterol; LDL: Low-Density Lipoprotein Cholesterol; GPT: Glutamate Pyruvate Transaminase Alanine Aminotransferase; CCI: Comorbidity Charlson Index; rMED Relative Mediterranean Score CVD: Cardiovascular Disease; Cr: Cancer deaths; DS: Digestive System deaths; DOC: Deaths from Other Cells reporting subject characteristics contain * Mean±(SD). **Median (IQR). ***Number. (Percentage) Percentages calculated per row.

Supplementary Table 3. Characteristics of Participants by Mediterranean Alcohol Drinking Pattern Score Categories. NUTRIHEP Study. Putignano (BA). Italy. 2005-2022

	Mediterranean Alcohol Drinking Pattern			
	High (7-9)	Moderate (4-6)	Low (0-3)	*p-value*
N***	716 (38.6)	1110 (59.7)	32 (1.7)	
Age at enrollment (yrs) *	50.37 (15.53)	44.44 (15.69)	48.33 (13.67)	<0.001
DBP (mmHg)*	127.31 (15.05)	123.33 (13.78)	129.84 (14.00)	<0.001
SBP (mmHg)*	80.95 (8.18)	78.80 (8.10)	82.81 (6.71)	<0.001
Weight (kg)*	70.32 (13.49)	69.70 (14.17)	82.37 (14.03)	<0.001
BMI (kg/m^2)*	26.04 (4.15)	25.71 (4.68)	28.33 (3.79)	0.002
Kcal days*	2017.86 (664.40)	2048.15 (725.45)	2838.01 (835.52)	<0.001
Triglycerides (mmol/L)*	1.33 (0.94)	1.21 (0.87)	1.65 (1.10)	0.002
Total Cholesterol (mmol/L)*	5.15 (0.99)	4.94 (1.05)	5.23 (0.74)	<0.001
HDL (mmol/L)*	1.36 (0.35)	1.35 (0.34)	1.26 (0.32)	0.19
LDL (mmol/L)*	3.20 (0.87)	3.04 (0.90)	3.26 (0.73)	<0.001
Glucose (mmol/L)*	5.72 (1.23)	5.54 (1.14)	6.20 (1.59)	<0.001
ALT (µkat/L)*	0.25 (0.13)	0.25 (0.18)	0.43 (0.30)	<0.001
Wine consumption (g alcohol/d) *	12.79 (10.95)	5.35 (11.66)	43.72 (19.46)	<0.001
Beer consumption (g alcohol/d) *	0.62 (1.11)	1.89 (4.00)	15.37 (11.70)	<0.001
Spirit consumption (g alcohol/d) *	0.88 (1.98)	1.13 (2.70)	10.00 (11.64)	<0.001
Sex***				
Female	378 (36.5)	655 (63.2)	3 (0.3)	<0.001
Male	338 (41.1)	455 (55.4)	29 (3.5)	
Smoke***				
Never/Former	617 (39.2)	937 (59.6)	19 (1.2)	<0.001
Current	99 (34.7)	173 (60.7)	13 (4.6)	
CCI**	2 (1-4)	1 (0-3)	3 (1-5)	<0.001
rMED**	9 (6-11)	9 (6-11)	6 (4-8.50)	0.003
Age at Death (yrs) **	67.15 (54.77-77.25)	59.51 (48.02-72.50)	65.57 (54.96-73.78)	<0.001
Observation time** (yrs)	16.66 (16.12-16.87)	16.70 (16.17-16.85)	16.59 (16.11-16.85)	0.95
Status***				
Alive and/or Censored	631 (37.7)	1.016 (60.7)	27 (1.6)	0.033
Dead	85 (46.2)	94 (51.1)	5 (2.7)	
Cause of Death***				
CVD	26 (50.0)	25 (48.1)	1 (1.9)	0.857
Cr	19 (48.7)	18 (46.2)	2 (5.1)	
DS	13 (46.4)	14 (50.0)	1 (3.6)	
DOC	27 (41.5)	37 (56.9)	1 (1.5)	
Education***				
Primary School	227 (45.2)	266 (53.0)	9 (1.8)	<0.001
Secondary School	205 (37.7)	326 (59.9)	13 (2.4)	
High School	214 (32.9)	427 (65.6)	10 (1.5)	
Graduated	70 (43.5)	91 (56.5)	0 (0.0)	
Job***				
Managers & Professionals	56 (37.6)	89 (59.7)	4 (2.7)	<0.001
Craft. Agricultural and Sales Workers	155 (36.4)	262 (61.5)	9 (2.1)	
Elementary Occupations	147 (36.6)	246 (61.2)	9 (2.2)	
Housewife	104 (36.5)	181 (63.5)	0 (0.0)	
Pensioneers	204 (51.8)	182 (46.2)	8 (2.0)	
Jobless	49 (24.6)	148 (74.4)	2 (1.0)	

Marital Status***				
Single	114 (27.4)	299 (71.9)	3 (0.7)	<0.001
Married/Coupled	571 (42.0)	760 (56.0)	27 (2.0)	
Separated/Divorced	6 (20.7)	22 (75.9)	1 (3.4)	
Widower	25 (45.5)	29 (52.7)	1 (1.8)	

DBP: Diastolic Blood Pressure; SBP: Systolic Blood pressure; BMI: Body Mass Index; HDL: High-Density Lipoprotein Cholesterol; LDL: Low-Density Lipoprotein Cholesterol; GPT: Glutamate Pyruvate Transaminase AlanineAminotransferase; CCI: Comorbidity Charlson Index; rMED Relative Mediterranean Score CVD: Cardiovascular Disease; Cr: Cancer deaths; DS: Digestive System deaths; DOC: Deaths from Other Cells reporting subject characteristics contain * Mean±(SD). **Median (IQR). ***Number. (Percentage) Percentages calculated per row.

Supplementary Table 4. Distribution of participants by scoring criteria of Mediterranean Alcohol Drinking Pattern score components.

		Mediterranean Alcohol Drinking Pattern		
	Score	Low	Moderate	High
Item		n (%)	n (%)	n (%)
Moderate alcohol intake (g/d)				
High intake: F>25 g/d; M>50 g/d	0	93 (26.8)	254 (73.2)	0 (0.0)
Low intake: F >0-5 g/d; M>0-10 g/d	1	0 (0.0)	930 (77.2)	274 (22.8)
Moderate intake: F 5-25 g/d; M 10-50 g/d	2	0 (0.0)	324 (17.4)	1.536 (82.6)
Alcohol consumption spread out over the week (d/week: week ratio)				
Not distributed:in Q1	0	93 (10.3)	480 (53.1)	331 (36.6)
Moderately distributed: in Q2-Q3	1	0 (0.0)	830 (42.6)	1.120 (57.4)
Evenly distributed: in Q4	2	0 (0.0)	198 (35.5)	359 (64.5)
Low spirit consumption (alcohol from spirit/total alcohol)				
High spirit consumption: ≥25%	0	1 (0.4)	242 (98.4)	3 (1.2)
Low spirit consumption: <25%	1	92 (2.9)	1.266 (40.0)	1.807 (57.1)
Wine preference (alcohol from wine/total alcohol)				
No wine preference: <75%	0	88 (5.6)	1.215 (77.0)	275 (17.4)
Wine preference ≥75%	1	5 (0.3)	293 (16.0)	1.535 (83.7)
Wine consumed preferably with meals (wine with meals/total wine)				
Out of meals <75%	0	0 (0.0)	0 (0.0)	0 (0.0)
Preferably with meals ≥75%	1	93 (2.7)	1.508 (44.2)	1.810 (53.1)
Preference for red wine over types of wine (red wine/total wine)				
No red wine preference <75%	0	12 (3.4)	308 (86.0)	38 (10.6)
Red wine preference ≥75%	1	81 (2.7)	1.200 (39.3)	1.772 (58.0)
No excess consumption (maximum drinks on a single occasion)				
Any excess: >5 drinks on a single occasion	0	91 (41.9)	126 (58.1)	0 (0.0)
No excess: ≤5 drinks on a single occasion	1	2 (0.1)	1.382 (43.3)	1.810 (56.7)

Table S5 Mortality Hazard Ratios (HR) and Subdistribution Hazard Ratios (SHR) according to the categories of the Mediterranean Alcohol-drinking pattern (MADP)

	MADP	
	Moderate (4-6)	Low (0-3)
	HR (95% CI)	HR (95% CI)
All causes Death	1.09 (0.89; 1.34)	1.46 (0.93; 2.28)
	SHR (95% CI)	SHR (95% CI)
CVD	1.08 (0.74; 1.58)	0.81 (0.30; 2.18)
Cr	1.06 (0.74; 1.51)	2.70**(1.51; 4.83)
DOC	1.17 (0.84; 1.62)	0.62 (0.23; 1.65)

High Adherence: referent category; **pvalue*<0.05 ***pvalue*<0.001. Model adjusted for sex (F vs M), Body Mass Index (<25 vs ≥25), Alanine Amine Transferase (<40 U/l vs ≥40 U/l), Triglycerides (<150 mg/L vs ≥150 mg/L), Comorbidity Charlson Index, smoking habits and Job. HR: Hazard Ratio; SHR: Subdistribution Hazard Ratio; CVD Cardiovascular Disease; Cr: Cancer DOC: Other Causes of Death

Table S6. Mortality Hazard Ratios (HR) and Subdistribution Hazard Ratios (SHR) according to the categories of the Relative Mediterranean Score without alcohol consumption (rMEDNA)

	rMEDNA	
	Moderate (4-6)	Low (0-3)
	HR (95% CI)	HR (95% CI)
All causes Death	1.13 (0.90; 1.43)	1.27 (0.96; 1.68)
	SHR (95% CI)	SHR (95% CI)
CVD	0.98 (0.65; 1.46)	0.83 (0.50; 1.46)
Cancer	1.49 (0.96; 2.30)	1.66(0.95; 2.58)
DOC	1.15 (0.77; 1.70)	1.37 (0.86; 2.18)

High Adherence: referent category; Model adjusted for sex (F vs M), Body Mass Index (<25 vs ≥25), Alanine Amine Transferase (<40 U/L vs ≥40 U/L), Triglycerides (<150 mg/L vs ≥150 mg/L), Comorbidity Charlson Index, smoking habits and Job. HR: Hazard Ratio; SHR: Subdistribution Hazard Ratio; CVD Cardiovascular Disease; Cr: Cancer DOC: Other Causes of Death

Table S7 Mortality Hazard Ratios (HR) and Subdistribution Hazard Ratios (SHR) according to the categories of the MADP+rMED No Alcohol

	All causes	CVD	Cancer	OCD
	HR (95% CI)	SHR (95% CI)	SHR (95% CI)	SHR (95% CI)
MADP#rMEDNA				
High High	1.00	1.00	1.00	1.00
High Moderate	1.24 (0.93; 1.65)	1.13 (0.69; 1.86)	1.44 (0.85; 2.48)	1.27 (0.82; 1.99)
High Low	1.26 (0.87; 1.22)	0.84 (0.43; 1.66)	1.27 (0.65; 2.48)	1.39 (0.81; 2.39)
Moderate High	1.37 (0.90; 2.08)	1.44 (0.70; 2.99)	1.06 (0.47; 2.37)	1.72 (0.89; 3.31)
Moderate Moderate	1.17 (0.82; 1.66)	1.01 (0.54; 1.89)	1.30 (0.69; 2.44)	1.31 (0.76; 2.25)
Moderate Low	1.50* (1.01; 2.23)	1.12 (0.54; 2.37)	1.86(0.95; 3.67)	1.40 (0.74; 2.66)
Low High	0.57 (0.08; 4.12)	1.72 (0.23; 12.9)	NE	NE
Low Moderate	1.78 (0.97; 3.27)	0.81 (0.17; 2.98)	4.10**(1.81; 9.27)	0.62 (0.15; 2.58)
Low Low	2.29* (1.04; 5.04)	0.77 (0.11; 5.47)	3.85*(1.29; 11.5)	1.24 (0.31; 4.89)

**pvalue*<0.05 ***pvalue*<0.001. Model adjusted for sex (F vs M), Body Mass Index (<25 vs ≥25), Alanine Amine Transferase (<40 U/L vs ≥40 U/L), Triglycerides (<150 mg/L vs ≥150 mg/L), Comorbidity Charlson Index, smoking habits and Job. HR: Hazard Ratio; SHR: Subdistribution Hazard Ratio; rMEDNA: Relative Mediterranean Score without alcohol consumption. CVD Cardiovascular Disease; OCD: Other Causes of Death; NE: Not Estimable

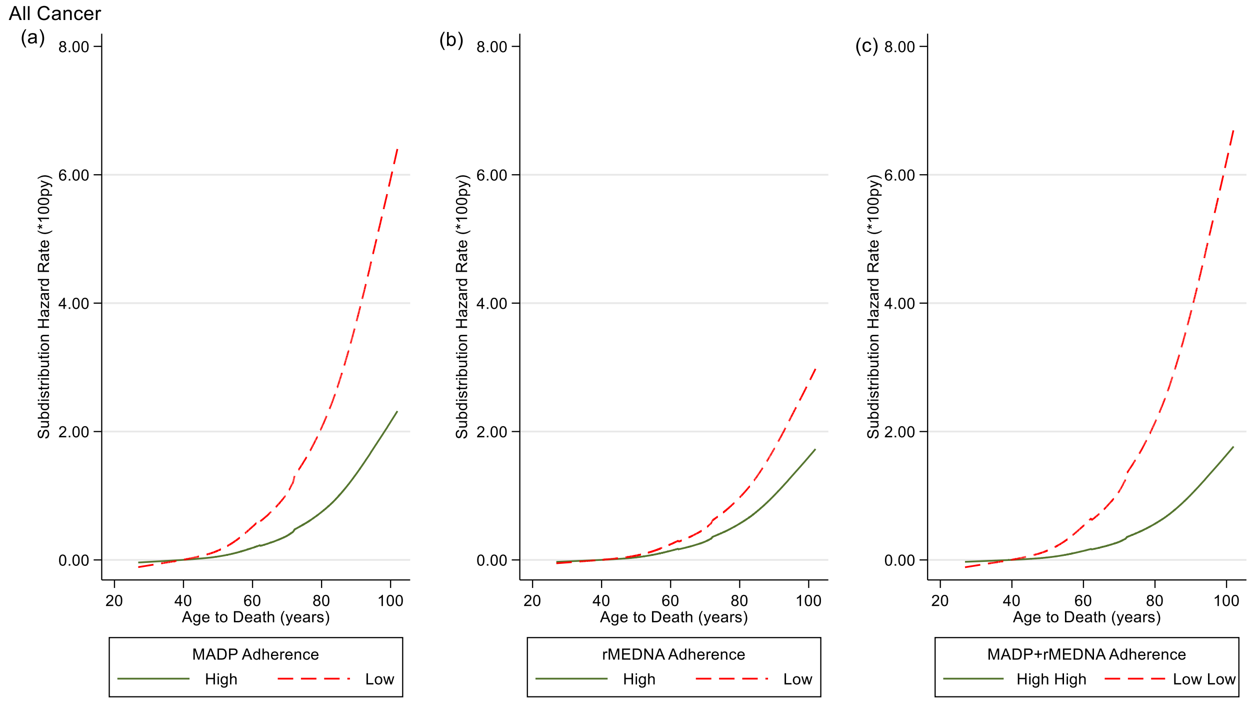

MADP:Mediterranean Alcohol Drinking Pattern; rMEDNA: Relative Mediterranean Diet Score without Alcohol consumption; py:person years

Figure S1: Subdistribution Hazard Rates for all causes of Death by (a) MADP, (b) rMEDNA, (c) MADP+rMEDNA
