# Supplementary figures and images for: High Adherence to a Mediterranean Alcohol-Drinking Pattern and Mediterranean Diet Can Mitigate the Harmful Effect of Alcohol on Mortality Risk
Source: Nutrients. 2023 Dec 24;16(1):59. doi: 10.3390/nu16010059 (PMC10780794; doi:10.3390/nu16010059)

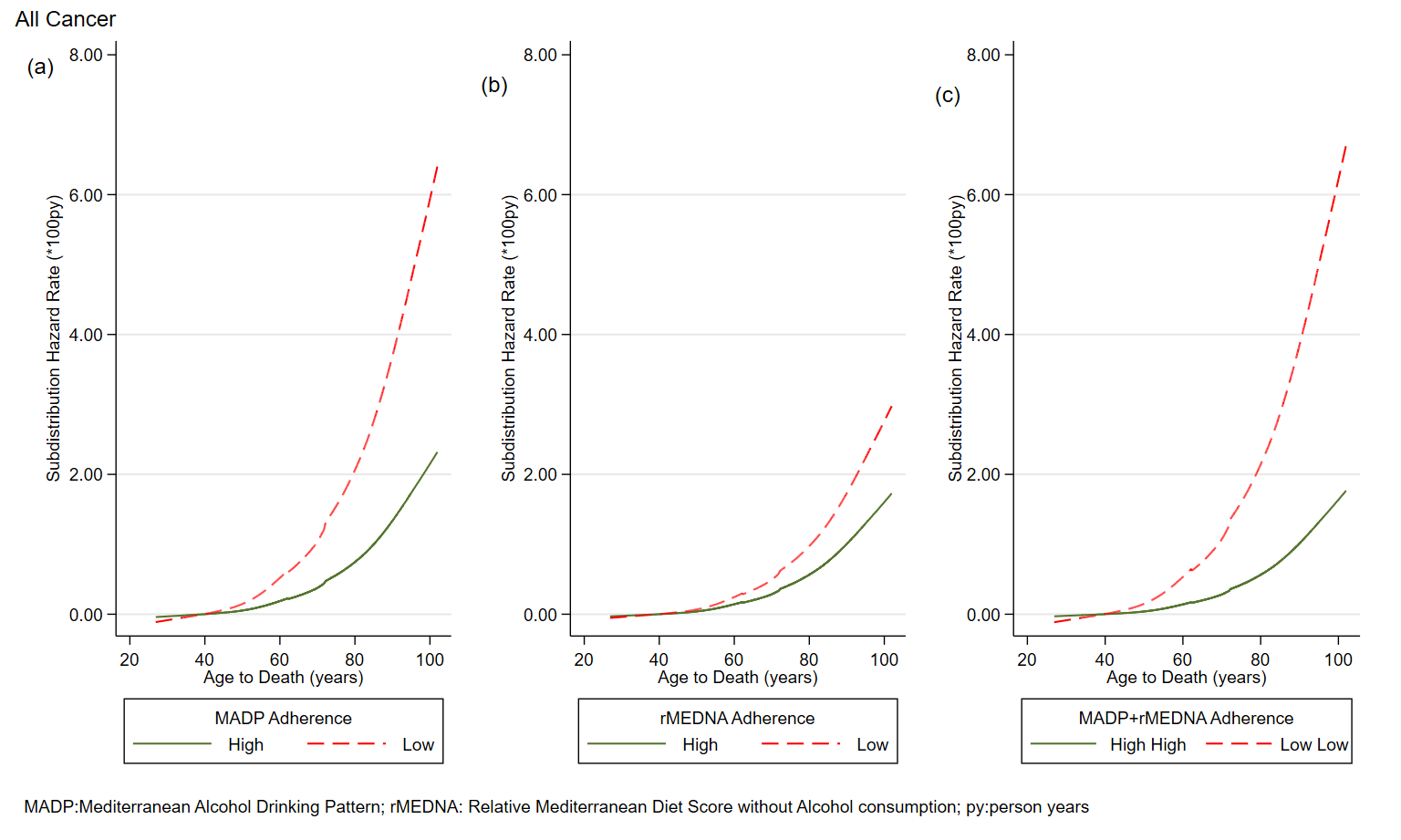

Supplement: Supplementary file 1 [file nutrients-16-00059-s001.zip › Figure S1.tif]
